# The Effect of Swallowing Action Observation Therapy on Resting fMRI in Stroke Patients with Dysphagia

**DOI:** 10.1155/2023/2382980

**Published:** 2023-04-21

**Authors:** Ming Zeng, Zhongli Wang, Xuting Chen, Meifang Shi, Meihong Zhu, Jingmei Ma, Yunhai Yao, Yao Cui, Hua Wu, Jie Shen, Lingfu Xie, Jianming Fu, Xudong Gu

**Affiliations:** ^1^Department of Rehabilitation Medicine, The Second Affiliated Hospital of Jiaxing University, The Second Hospital of Jiaxing City, Jiaxing, Zhejiang Province 314000, China; ^2^Department of Physical Therapy, Beijing Bo'ai Hospital, China Rehabilitation Research Center, Capital Medical University School of Rehabilitation Medicine, Beijing 100068, China; ^3^First Clinical Medical College, Nanchang University, Nanchang, Jiangxi Province 330031, China

## Abstract

**Objective:**

Many stroke victims have severe swallowing problems. Previous neuroimaging studies have found that several brain regions scattered in the frontal, temporal, and parietal lobes, such as Brodmann's areas (BA) 6, 21, and 40, are associated with swallowing function. This study sought to investigate changes in swallowing function and resting-state functional magnetic resonance imaging (rs-fMRI) in stroke patients with dysphagia following action observation treatment. It also sought to detect changes in brain regions associated with swallowing in stroke patients.

**Methods:**

In this study, 12 healthy controls (HCs) and 12 stroke patients were recruited. Stroke patients were given 4 weeks of action observation therapy. In order to assess the differences in mfALFF values between patients before treatment and HCs, the fractional amplitude of low-frequency fluctuations (fALFF) in three frequency bands (conventional frequency band, slow-4, and slow-5) were calculated for fMRI data. The significant brain regions were selected as regions of interest (ROIs) for subsequent analysis. The mfALFF values were extracted from ROIs of the three groups (patients before and after treatment and HCs) and compared to assess the therapeutic efficacy.

**Results:**

In the conventional band, stroke patients before treatment had higher mfALFF in the inferior temporal gyrus and lower mfALFF in the calcarine fissure and surrounding cortex and thalamus compared to HCs. In the slow-4 band, there was no significant difference in related brain regions between stroke patients before treatment and HCs. In the slow-5 band, stroke patients before treatment had higher mfALFF in inferior cerebellum, inferior temporal gyrus, middle frontal gyrus, and lower mfALFF in calcarine fissure and surrounding cortex compared to HCs. We also assessed changes in aberrant brain activity that occurred both before and after action observation therapy. The mfALFF between stroke patients after therapy was closed to HCs in comparison to the patients before treatment.

**Conclusion:**

Action observation therapy can affect the excitability of certain brain regions. The changes in brain function brought about by this treatment may help to further understand the potential mechanism of network remodeling of swallowing function.

## 1. Introduction

One of the most typical causes of neurological disability in adults is stroke, and in some countries, it is also the leading cause of mortality. In the last ten years, the incidence and prevalence of stroke have increased. Every year, more than 2 million new stroke patients are diagnosed in China. Because of the aging population, the high prevalence of risk factors such as high blood pressure, and poor management, this burden is likely to grow even more [1]. Dysphagia is a serious after-stroke complication that increases the risk of dehydration, malnutrition, aspiration pneumonia, and death [2]. According to an epidemiological survey, dysphagia affected 51.14 percent of the 2872 hospitalized stroke patients [3].

However, treatments for dysphagia caused by stroke are still limited. Traditional treatments include alternative feeding methods, dietary adjustment, behavioral and postural techniques, peripheral sensory and motor stimulation techniques, or active exercise designed to enhance the function of selected muscle groups and shorten exercise time at the beginning of swallowing [4, 5]. These techniques are designed to ensure the safety and proper food intake of stroke patients with dysphagia. Although these techniques have achieved some success, the effective recovery of dysphagia after stroke remains a challenge [2].

Swallowing is a complex sensory movement that involves the interaction between the cerebral cortex and subcortical nerves. The rehabilitation of swallowing function in stroke patients is impacted by a variety of factors, including the location of brain injury and the activation of brain swallowing-related cortical neuronal networks during swallowing [6]. Because the actual situation of stroke patients varies greatly, it is very important to set specific treatment plans for different patients; this makes treating dysphagia challenging for all stroke rehabilitation teams.

In recent years, due to the discovery of mirror neuron (MN) and ongoing research, a new rehabilitation method for stroke dysphagia has been developed. MN is a special type of neuron that triggers when one person performs an action or notices another person performing an action with a similar purpose [7]. The action observation therapy used in this study is based on this theory, but there is no completely unified discussion on the specific distribution of MN in the brain. The action observation therapy is a new method of rehabilitation which has become increasingly popular in recent years. Patients can improve their function by carefully observing the relevant motion videos and trying to imitate them. It is widely used in the study of recovery of poststroke dysfunction, such as swallowing and limb function [8].

Resting functional magnetic resonance imaging (fMRI) measures blood oxygen level-dependent (BOLD) signals, which are used to measure the spontaneous fluctuations of the brain. It has been widely used in Parkinson's disease [9, 10], Alzheimer's disease [11, 12], stroke [13, 14], and other diseases. Park et al. [15] found that in contrast to healthy people, stroke patients had decreased connection in the contralateral M1 and occipital cortex and increased functional connectivity in the bilateral thalamus, cerebellum, and ipsilateral frontal and parietal cortex. Additionally, it was discovered that motor recovery half a year after stroke was favorably connected with the functional relationship between the ipsilateral M1 area and contralateral thalamus, auxiliary motor area, and middle frontal gyrus. The synchronization of brain activity between regions is reflected in functional connections. Local indicators have also been used in stroke research recently. ALFF [16] was proposed by Zang et al. in 2007, which measures the oscillation's amplitude at low frequencies. Based on this, fALFF has been enhanced. The calculation of fALFF [17] value was first proposed by Zou et al. Generally, by computing the proportion of a certain frequency band's power spectrum to its whole spectrum, one can determine the fALFF. This approach can enhance the sensitivity and specificity of detecting spontaneous brain activity in fMRI by suppressing the nonspecific signal components. In the study of Zhan et al. [18], the value of fALFF was calculated, and it was found that the values of fALFF in the cerebellum, anterior lobe, precentral gyrus, superior frontal gyrus, and parietal lobe increased after scalp acupuncture intervention. A potential clinical measure for predicting motor impairment is thought to be the fALFF value of the ipsilateral precentral and postcentral gyrus. Researchers discovered that low-frequency oscillations (LFOs) with a frequency range of 0.073-0.025 Hz (slow-3: 0.073-0.198 Hz, slow-2: 0.198-0.25 Hz) are frequently found within the white matter and LFOs with a frequency range of 0.01-0.073 Hz (slow-4: 0.027-0.073 Hz, slow-5: 0.01-0.027 Hz) embody the spontaneous activities of neurons in the gray matter [19]. Prior research [20] largely concentrated on the variation of fALFF in conventional frequency band. However, no research has focused on whether there is band specificity in the brain regions that differ between stroke patients and healthy people in multifrequency bands. Therefore, this study intends to use rs-fMRI to conduct a multiband analysis of regional brain movement in healthy people and stroke patients, to find out the possible relationship between MN and action observation therapy from the mechanism level, and to evaluate the therapeutic effect of action observation therapy more comprehensively and deeply.

## 2. Materials and Methods

### 2.1. Participants

**Figure 1:**
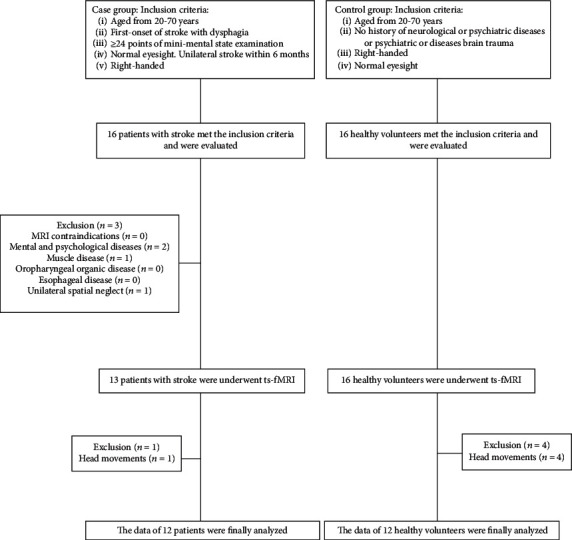
A schematic illustration of the participant selection process used in the present study.

**Figure 2:**
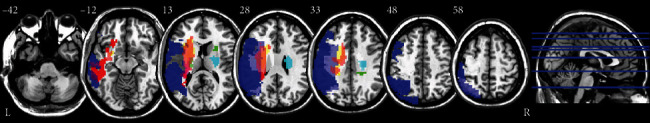
Focal area overlap map in stroke patients.

From June 2019 to January 2022, a case-control observational study was carried out in the Second Hospital of Jiaxing (Zhejiang, China). In this study, 12 stroke patients and 12 healthy controls (HCs) who were matched for age and gender were recruited. The inclusion criteria were in line with the latest stroke diagnostic criteria [21], and the patients with dysphagia were confirmed by swallowing angiography after the first stroke. The following were some of the patient exclusion criteria: (1) MRI contraindications, (2) mental and psychological diseases, (3) muscle disease, (4) oropharyngeal organic disease, (5) esophageal disease, and (6) unilateral spatial neglect. Each subject was assessed by general condition assessment and swallowing function assessment; the former includes Barthel index [22], Nutritional Risk Screening 2002 (NRS2002) [23], and John Hopkins Fall Risk Assessment Tool (JHFRAT) [24], while the latter was assessed by Eating Assessment Tool-10 (EAT-10) [25]. The Ethics Committee of the Second Affiliated Hospital of Jiaxing University approved this study (no. jxey-2018SKZ03), and it was conducted in accordance with the ethical guidelines outlined in the Declaration of Helsinki. A formal informed consent form was signed by each participant. The China Clinical Trial Registry has this trial listed (ChiCTR1900021849).  Figure 1 depicts the detailed enrolling process; Figure 2 depicts the lesion area in 12 stroke patients; Tables 1 and 2 depict the detailed participant characteristics.

### 2.2. Action Observation Therapy

We recorded a video of swallowing action observation of 7 min, which was used to observe the swallowing action of patients. The video includes the following: (a) swallow solid food and observe models chewing and swallowing rice from the front; (b) swallow solid food and observe the model chewing and swallowing an apple directly; (c) swallow solid food and observe the model chewing and swallowing an apple from the side; (d) swallow liquid food and observe the model swallowing yogurt directly; (e) empty swallowing and observe the model's imitation of swallowing three mouthpieces without drinking water (in (a), (b), (c), (d), and (e) swallowing videos, the model will properly raise her head and swallow during the process of chewing and swallowing and zoom in to show the swallowing movements of the model's mouth and throat); (f) facial muscle movement, positive observation of the model to do gill exercise; (g) neck movement, positive observation of the model's neck movement; (h) tongue muscle movement, positive observation of the model before and after the tongue stretching action; (i) tongue muscle resistance movement, observe the model's tongue resistance movement before and after stretching tongue, and use tongue depressor to exert resistance to the model's tongue muscle movement; (j) lip muscle movement, observe the lip movement of the model when making the sound of “e, u, and o”; and (k) lip muscle resistance action, front observation of the model with her lips on the tongue depressor and then against another person to remove the tongue depressor.

The single treatment time of action observation therapy was 10 minutes, once a day, five days a week for four weeks. In addition, each patient in the study received conventional swallowing therapy, including sensory stimulation, lingual resistance training, swallowing position placement, supraglottic swallowing, and Mendelson swallowing, for 30 minutes at a time, once a day, five days a week for four weeks.

### 2.3. MRI Data Acquisition

All stroke patients completed two MRI examinations (before and after treatment), while healthy controls completed only one MRI examination. The MRI was performed using a 3.0 T superconducting MRI equipment with a typical 32-channel head coil (Philips, Netherlands). The scanning sequences included initial positioning image, high-resolution T1-weighted structural image for segmentation, and registration. Every participant was once again instructed to unwind with their eyes closed, breathe deeply, and minimize head movements and cognitive processes. The use of sponge cushions helped to minimize head motion. Earplugs made of rubber were employed to block out scanner noise.

The following scan parameters were used to acquire anatomical pictures using a high-resolution T1-weighted 3-dimensional fast gradient echo sequence: 170 slices in sagittal position, slice thickness = 1 mm, slice gap = 0 mm, repetition time (TR) = 7.9 ms, echo time (TE) = 3.5 ms, flip angle = 8°, field of view (FOV) = 256 × 256 mm^2^, acquisition matrix = 256 × 256, voxel size = 1 × 1 × 1 mm^3^, and total scanning time = 5 min and 2 s.

Fast spin echo sequence uses the following parameters for conventional T2-weighted imaging to validate the lesion's location: 24 slices in axial transverse position, slice thickness = 5 mm, gap = 6 mm, TR = 3000 ms, TE = 80 ms, flip angle = 90°, FOV = 230 × 190 mm^2^, acquisition matrix = 328 × 224, voxel dimensions = 0.4 × 0.4 × 6 mm^3^, and total scanning time = 1 min and 30 s.

The following are the resting-state functional magnetic resonance data using echo-planar imaging (EPI) sequence: 46 slices in transverse section, slice thickness = 2.5 mm, slice gap = 0.5 mm, TR = 2000 ms, TE = 20 ms, flip angle = 90°, FOV = 240 × 240 mm^2^, acquisition matrix = 96 × 96, and voxel dimensions = 2.5 × 2.5 × 3 mm^3^. A skilled radiologist determined the volume of each patient's lesion. Using MRIcron software (http://www.mricro.com), the contour of the aberrant signal in the T2-weighted image was manually drawn layer by layer, and the T2-weighted image space was standardized to the Montreal Neurological Institute (MNI) space. Each patient's lesion mask was added together to create a lesion overlay, which was then placed on the MRIcron standard template. For all stroke patients, the final lesion overlays were displayed by layer.

### 2.4. fMRI Data Processing

Statistical Parametric Mapping 12 (SPM12) and RESTPlus version 1.25 were used to process all functional imaging data. To reduce the nonequilibrium effect of magnetization, in order to adapt to the surroundings, the first 10 time points were eliminated. Slice timing was applied to the remaining images for each individual (the number of time layers was 46 and the scanning sequence was interlayer scanning), realignment (participants whose maximum head movement was larger than 3 mm and 3 degrees were excluded), segmentation, and coregistration with the subjects' own structural pictures. The Montreal Neurological Institute (MNI) space was used to normalize all the aligned functional pictures, and the standardized voxel size is 3 × 3 × 3mm^3^ and spatial smoothing of standardized functional images using Gaussian kernel 6 × 6 × 6 mm; after removing the linear trend, the Friston-24 motion parameters, white matter, and cerebrospinal fluid signal were regressed out using detrending.

The fALFF was calculated for the preprocessed data. The frequency bands are, respectively, set to the traditional frequency bands (0.01-0.08 Hz), slow-4 (0.027-0.073 Hz), and slow-5 (0.01-0.027 Hz). Subsequent statistical analysis uses the mfALFF values. We followed the methods of Zhou et al. [26].

### 2.5. Statistical Analysis

In this study, age and course data were analyzed using the nonparametric test, gender data were analyzed using the Pearson chi-square test, and demographic characteristics were statistically analyzed using SPSS 25.0 software.

By using a two-sample *T*-test, the values of mfALFF in three frequency bands were compared between HCs and patients before treatment, and age, location of lesion, education level, and so on were taken as covariates. The difference in brain areas between the two groups was determined using the Gaussian random field (GRF) multiple comparison correction method, voxel *P* < 0.005, cluster *P* < 0.05. These several brain areas served as the ROI for the analyses that followed.

For each cluster survived after GRF corrected, the mean mfALFF value was extracted for every participant. Two-sample *T*-test was used for patients and HCs, and paired *T*-test was used for patients before and after treatment. Pearson correlation coefficients were calculated to evaluate the correlations between the change rate of regional brain index (mfALFF) and the change rate of swallowing function score in stroke patients before and after treatment. The threshold value was *P* < 0.05.

## 3. Results

### 3.1. Demographic and Clinical Characteristics

Tables 1 and 2 analyze the demographic information and clinical effectiveness of the three groups. There was no discernible gender difference between the patients before treatment and HCs, but there were statistical differences in age, course of disease, stroke classification, lesion site, education years, Barthel index, NRS2002, JHFRAT, and EAT-10. The EAT-10 score of the patients after treatment was lower than that before treatment.

### 3.2. Results of mfALFF Analysis

**Figure 3:**
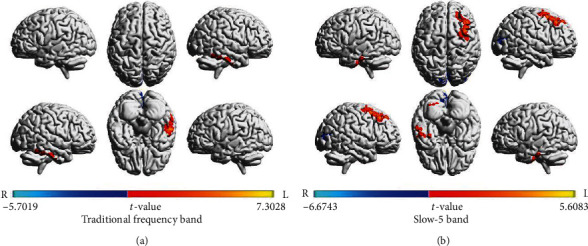
Difference of brain regions in three frequency bands between patients before treatment and HCs.

In the traditional frequency band, compared with HCs, the mfALFF of inferior temporal gyrus increased, and the calcarine fissure and surrounding cortex and thalamus decreased in stroke patients before treatment. In the slow-4 band, we did not find significant differences in mfALFF values between stroke patients before treatment and HCs. In the slow-5 band, compared with HCs, the mfALFF of inferior cerebellum, inferior temporal gyrus, and middle frontal gyrus increased, while calcarine fissure and surrounding cortex decreased in stroke patients before treatment (Table 3 and Figure 3).

### 3.3. Effect of Action Observation Therapy

**Figure 4:**
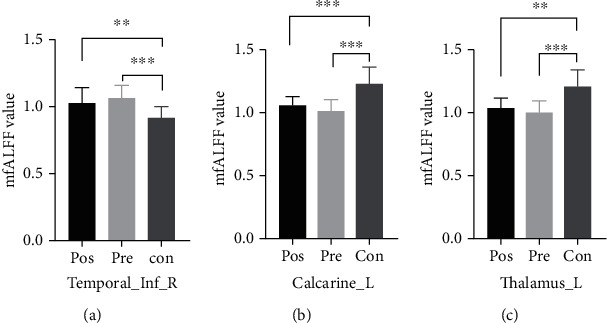
The difference of mfALFF in traditional frequency band between patients before and after treatment and HCs (pos: stroke patients after treatment; pre: stroke patients before treatment; con: healthy controls; ^∗∗^ indicated *P* < 0.01; ^∗∗∗^ indicated *P* < 0.001).

**Figure 5:**
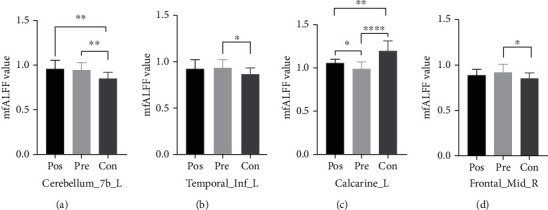
The difference of mfALFF in slow-5 band between patients before and after treatment and HCs (pos: stroke patients after treatment; pre: stroke patients before treatment; con: healthy controls; ^∗^ indicated *P* < 0.05; ^∗∗^ indicated *P* < 0.01; ^∗∗∗∗^ indicated *P* < 0.0001).

In the ROIs detected in the traditional frequency band, the difference of mfALFF between the patients after treatment and the HCs was less than that before treatment, mainly in the following brain regions: inferior temporal gyrus and thalamus. In slow-4 frequency band, no effective ROIs were found. In the ROIs detected in the slow-5 band, the difference of mfALFF between the patients after treatment and the HCs was less than that before treatment, mainly in the following brain regions: inferior temporal gyrus, calcarine fissure and surrounding cortex, and middle frontal gyrus (Tables 4 and 5 and Figures 4 and 5).

### 3.4. Correlation Analysis

**Figure 6:**
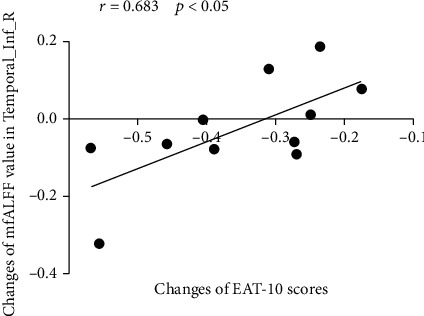
Analysis of the correlation between the change rate of mfALFF and swallowing function score in the inferior temporal gyrus.

In this study, correlation analysis was conducted between the change rate of regional brain index (mfALFF) and the change rate of swallowing function score in stroke patients before and after treatment. The results showed that there was a significant correlation between the change rate of mfALFF in inferior temporal gyrus and the change rate of swallowing function score; other ROIs failed to suggest that there was a significant correlation between the change rate of mfALFF and the change rate of swallowing function score (Table 6 and Figure 6).

## 4. Discussion

In the multiband detection of this study, it was found that action observation therapy could significantly reduce the difference of mfALFF between patients after treatment and HCs compared with that before treatment between patients and HCs. It is mainly distributed in the following brain regions: inferior temporal gyrus, calcarine fissure and surrounding cortex, thalamus, and middle frontal gyrus, most of which are independent of the lesions. There is also partial overlap between a small part of the brain area and the focus. In addition, we also found that there was a significant correlation between the change rate of mfALFF in the inferior temporal gyrus region and the change rate of swallowing function score before and after action observation therapy. All these results suggest that action observation therapy can indeed improve the swallowing function of stroke patients and change the excitability of brain areas related to swallowing.

Action observation therapy is a new method of rehabilitation, which is mostly used in exercise and speech rehabilitation [27–29]. The theoretical mechanism is believed to be related to MN, which are unique neurons that are activated when one person performs an action and watches another person perform a similar action [7]. Some scholars believe that there is also a MN system related to swallowing in the human brain. Ushioda et al. [30] employed magnetoencephalography to assess the active regions of the cerebral cortex connected to the MN system during swallowing in 10 healthy participants. The experimental group received visual and auditory swallowing stimulation, while the control group received static frame images and artificial sound. It was found that BA6 (premotor cortex and supplementary motor area) and BA40 (superior marginal gyrus) corresponding to the MN system were active between 620 and 720 milliseconds before drinking water was triggered. Jing et al. used task-stated fMRI to identify areas of the brain that were active during observation and swallowing in healthy subjects [31]. They discovered that the MN system, along with the left BA21 (middle temporal gyrus) and left BA6 (supplementary motor area), was engaged in the observation and execution of swallowing movements. However, from the observations obtained in this study alone, we were not able to find strong support for the role of MN in the relevant brain regions, but this does not exclude the possibility that mirror neurons play a role in the swallowing process, and the unsatisfactory results of the study may be influenced by various factors, including the persistent effect of the action observation therapy and individual differences in the site of lesion of the subjects.

There is also some evidence that the temporal lobe is associated with swallowing. Hamdy et al. [32] found that there is a significant asymmetry in the swallowing center between the hemispheres during volitional swallowing, and this recruitment phenomenon can involve the temporal lobe, the primary sensorimotor cortex, and other brain regions. The temporal lobe is believed by PET researchers to be crucial in helping people recognize the quality of their tastes. The prefrontal brain and temporal lobe may be involved in the control of swallowing [33]. The inferior temporal gyrus is connected to taste and appearance of food and may promote swallowing and diet control. Toogood et al. [34] suggested that different stages of swallowing are mediated by different regions. For example, a significant increase in activation in several regions, including bilateral thalamus, can be observed during swallowing preparation, while the increase in excitability during swallowing is mainly in the insular and dorsolateral peripheral cortex. Similarly, Martin et al. [35] found that during active tongue lifting, activation of related brain regions such as the thalamus and precentral/postcentral gyrus increased and that specific areas would mediate the occurrence and execution of a specific swallowing process. In addition, the middle frontal gyrus is also thought to be involved in continuous exercise programs, especially swallowing [36], which is active after swallowing water or saliva [37]. According to the above studies, we speculate that action observation therapy mainly affects the preparation stage of swallowing, including passive observation and unconscious simulation of swallowing movement. Thus, it promotes the activation of a series of brain regions, including the inferior temporal gyrus, middle frontal gyrus, and subcortical nuclei, promotes the swallowing compensatory function in the cortical and subcortical areas, and finally achieves the purpose of improving the swallowing function.

In addition, we also observed signal differences in some cerebellar regions between stroke patients before treatment and HCs, although we did not find the practical significance of cerebellum in the recovery of swallowing function in this study. However, there are still a large number of studies that support the cerebellum to actively participate in the regulation of human swallowing [32]. In early functional neuroimaging studies, chewing, oral and facial movement, lip and tongue movement, and whistling have been shown to activate the cerebellar hemisphere [38]. The cerebellum plays a role in the regulation of supplementary swallowing mechanism. This may explain how dysphagia manifests as delayed swallowing, malfunction of the esophageal sphincter, etc. We think this is also a research direction worth digging.

At the same time, this study also has some shortcomings: first, because the sample size is small and the subjects are not classified, the results of this study are only used as a preliminary exploratory study. Secondly, we only use a single resting-state fMRI to analyze the brain areas of MN, excluding EEG and motor-evoked potentials for further analysis. In order to better understand the impact of exercise observation therapy on the swallowing network, we therefore plan to examine the disease's subtypes on a large sample in the future and select a variety of detection methods to represent changes in brain excitability.

## 5. Conclusions

Using fALFF, we found that there was band specificity in the different brain regions between patients and healthy people before treatment; that is, the spatial distribution was different in different frequency bands. Observation of action therapy may improve the abnormal brain activity of patients to some extent and assist patients receive better care.

## Figures and Tables

**Table 1:** Demographic and clinical characteristics of stroke patients and HCs.

Group	Course of disease (day)	Stroke classification		Lesion site		
		Cerebral infarction	Cerebral hemorrhage	Lateral ventricle	Frontal/temporal/parietal lobe	Basal ganglia
Healthy controls						
Stroke patients	34.25 ± 16.43^∗^	9^∗^	3^∗^	2^∗^	4^∗^	6^∗^

The stroke classification and lesion site between the two groups were compared using the chi-square test, and the course of disease was compared using the independent two-sample *T*-test. ^∗^*P* < 0.001, there was a statistical difference between the two groups.

**Table 2:** Demographic and clinical characteristics of stroke patients and HCs.

	Healthy controls (*n* = 12)	Stroke patients (*n* = 12)	Test statistic	*P* value
Age (year)	51.17 ± 13.02	66.08 ± 10.01	-3.147	0.005
Gender (male/female)	6/6	8/4	0.685	0.4076
Edu years (year)	12.08 ± 4.19	6.00 ± 1.81	4.619	<0.001
*General function*				
Barthel index	100	31.67 ± 18.13	13.055	<0.001
Nutritional Risk Screening 2002 (NRS2002)	0	1.83 ± 1.59	-4.004	<0.001
John Hopkins Fall Risk Assessment Tool (JHFRAT)	0	10.50 ± 4.03	-9.017	<0.001
*Swallowing function*				
Eating Assessment Tool-10 (EAT-10)	0	Pretreatment 32.00 ± 4.45	-24.901	<0.001
		Posttreatment 20.58 ± 4.46	8.486	<0.001

The gender distribution between the two groups was compared using the chi-square test, and the age, education year, Barthel index, NRS2002, JHFRAT, and EAT-10 scores were compared using the independent two-sample *T*-test. A paired *T*-test was used to compare the EAT-10 scores for the stroke group before and after treatment.

**Table 3:** Difference in brain regions of mfALFF between stroke patients before treatment and healthy controls.

Brain area	Voxel size	MNI coordinates	Peak *T*-value
*Conventional band (0.01-0.08 Hz)*			
Temporal_Inf_R	72	60 -21 -30	7.3028
Calcarine_L	145	-9 -87 6	-5.7019
Thalamus_L	49	-6 -15 12	-4.9552
*Slow-4 band: 0.027-0.073*			
NA			
*Slow-5 band: 0.01-0.027*			
Cerebellum_7b_L	54	-24 -75 -54	5.1098
Temporal_Inf_L	50	-33 -9 -45	5.6083
Calcarine_L	672	-12 -87 9	-6.6743
Frontal_Mid_R	89	42 30 45	5.5876

*T*: statistical value of mfALFF differences between the two groups (negative values: patients before treatment<healthy controls; positive values: patients before treatment>healthy controls); MNI: Montreal Neurological Institute Coordinate System or Template; Temporal_Inf_R: inferior temporal gyrus; Calcarine_L: calcarine fissure and surrounding cortex; Thalamus_L: thalamus; Cerebellum_7b_L: inferior cerebellum; Frontal_Mid_R: middle frontal gyrus.

**Table 4:** The mfALFF values of these clusters were extracted for every participant and compared between each pair of the three groups in traditional band.

MNI coordinates	Brain region	Contrast	*T*-value	*P* value
60 -21 -30	Temporal_Inf_R (aal)	pre vs. con	4.351	0.0003
		pos vs. pre	0.7371	0.478
		pos vs. con	2.961	0.0075

-9 -87 6	Calcarine_L (aal)	pre vs. con	4.64	0.0001
		pos vs. pre	1.103	0.2959
		pos vs. con	4.028	0.0006

-6 -15 12	Thalamus_L (aal)	pre vs. con	3.876	0.0009
		pos vs. pre	0.5864	0.5706
		pos vs. con	3.158	0.0047

Temporal_Inf_R (aal): inferior temporal gyrus; Calcarine_L (aal): calcarine fissure and surrounding cortex; Thalamus_L (aal): thalamus.

**Table 5:** The mfALFF values of these clusters were extracted for every participant and compared between each pair of the three groups in slow-5 band.

MNI coordinates	Brain region	Contrast	*T*-value	*P* value
-24 -75 -54	Cerebellum_7b_L (aal)	pre vs. con	2.887	0.0088
		pos vs. pre	0.3407	0.7404
		pos vs. con	2.896	0.0086

-33 -9 -45	Temporal_Inf_L (aal)	pre vs. con	2.097	0.0483
		pos vs. pre	0.3555	0.7296
		pos vs. con	1.528	0.1415

-12 -87 9	Calcarine_L (aal)	pre vs. con	4.865	<0.0001
		pos vs. pre	2.333	0.0418
		pos vs. con	3.743	0.0012

42 30 45	Frontal_Mid_R (aal)	pre vs. con	2.587	0.0172
		pos vs. pre	1.059	0.3145
		pos vs. con	1.874	0.0749

Cerebellum_7b_L (aal): inferior cerebellum; Temporal_Inf_L (aal): inferior temporal gyrus; Calcarine_L (aal): calcarine fissure and surrounding cortex; Frontal_Mid_R (aal): middle frontal gyrus.

**Table 6:** The correlation between the change rate of regional brain index (mfALFF) and swallowing function score in stroke patients before and after treatment.

Brain area	*r*-value	*P* value	Significant
Temporal_Inf_R (60 -21 -30)	0.683	0.0205	^∗^
Calcarine_L (-9 -87 6)	-0.4467	0.1684	ns
Thalamus_L (-6 -15 12)	0.4112	0.209	ns
Cerebellum_7b_L (-24 -75 -54)	-0.1597	0.6391	ns
Temporal_Inf_L (-33 -9 -45)	0.1228	0.7191	ns
Calcarine_L (-12 -87 9)	-0.299	0.3718	ns
Frontal_Mid_R (42 30 45)	0.1795	0.5974	ns

^∗^There was a correlation between the change rate of mfALFF and the change rate of swallowing function score; ns: there was no significant correlation between the change rate of mfALFF and the change rate of swallowing function score.

## Data Availability

The data used to support the findings of this study are available from the corresponding authors upon reasonable request.
